# Activation of Innate Immunity by Therapeutic Nucleic Acids

**DOI:** 10.3390/ijms222413360

**Published:** 2021-12-12

**Authors:** Ali Bishani, Elena L. Chernolovskaya

**Affiliations:** Institute of Chemical Biology and Fundamental Medicine, Siberian Branch of the Russian Academy of Sciences, Acad. Lavrentiev Avenue 8, 630090 Novosibirsk, Russia; ali1bishani@gmail.com

**Keywords:** immunotherapy, innate immunity, therapeutic nucleic acids, pattern recognition receptors, immunostimulating RNA, CpG-oligonucleotides

## Abstract

Nucleic acid-based therapeutics have gained increased attention during recent decades because of their wide range of application prospects. Immunostimulatory nucleic acids represent a promising class of potential drugs for the treatment of tumoral and viral diseases due to their low toxicity and stimulation of the body’s own innate immunity by acting on the natural mechanisms of its activation. The repertoire of nucleic acids that directly interact with the components of the immune system is expanding with the improvement of both analytical methods and methods for the synthesis of nucleic acids and their derivatives. Despite the obvious progress in this area, the problem of delivering therapeutic acids to target cells as well as the unresolved issue of achieving a specific therapeutic effect based on activating the mechanism of interferon and anti-inflammatory cytokine synthesis. Minimizing the undesirable effects of excessive secretion of inflammatory cytokines remains an unsolved task. This review examines recent data on the types of immunostimulatory nucleic acids, the receptors interacting with them, and the mechanisms of immunity activation under the action of these molecules. Finally, data on immunostimulatory nucleic acids in ongoing and completed clinical trials will be summarized.

## 1. Introduction

Immunotherapy is the treatment of disease through the manipulation of immune responses by either amplification or suppression. During recent decades, immunotherapy has gained increased attention from researchers [1], especially in the fields of cancer and infectious diseases [2]. Several immunotherapeutic approaches against cancer, including specific and nonspecific immunotherapy, were tested in clinical trials, and some drugs have already been introduced into clinical practice. Major immunotherapeutic approaches include the use of monoclonal antibodies, immune checkpoint inhibitors, and cytokines [3,4,5]. Preparations based on monoclonal antibodies have been successfully proven for the treatment of tumoral diseases: in 2021, 35 years after the FDA approved the first monoclonal antibody, the 100th monoclonal antibody product was approved [6]. If molecular targets for the particular diseases have not been identified, nonspecific immunity activation could provide antitumor, antimetastatic, and antiviral effects. However, each of these methods still has side effects that impede the successful completion of clinical trials and their approval for use in clinical practice. Tolerated side effects of approved drugs often complicate treatment and require concomitant medication support and subsequent rehabilitation [7,8]. Such problems arising from the use of immunotherapy have attracted the attention of researchers to the study of immunity activators using natural mechanisms of action, particularly immunostimulatory nucleic acids.

Immunostimulatory nucleic acids, which are used in immunotherapy, induce cytokine and interferon synthesis and secretion. Naturally occurring exogenous nucleic acids are usually a sign of invading viruses and bacteria and initiate an innate immune response, which will help cells to eliminate the threat and alert neighboring cells. This gave rise to the idea of using synthetic nucleic acids to manipulate the immune response: poly(I:C), the analog of dsRNA, and CpG oligonucleotides have been widely used in recent decades to study the innate response in vivo and in vitro [9]. During experimentation with short double-stranded RNA sequences for RNA interference, it was noticed that different sequences induced an immune response to varying levels, and this also helped researchers to choose the sequences that induced the strongest response and use them to check immunomodulatory activity.

Naked nucleic acids cannot efficiently enter cells by themselves and are not stable inside cells due to nuclease degradation; therefore, delivery systems are required for their application. Different types of delivery vehicles, such as nanoparticles and cationic liposomes, were developed for this purpose [10,11], but this is not a risk-free procedure and some obstacles appeared, such as the toxicity of lipoplexes and the difficulty of finding specific biomarkers to make the delivery system more specific. The problems of delivering therapeutic nucleic acids to target cells have been described in numerous reviews [12,13,14].

Immunotherapy (including the use of immunostimulatory nucleic acids) has its own advantages and disadvantages. The main advantage is that it works effectively in some malignancies that are refractory to chemotherapy and radiotherapy, and thus expands the choice of treatment options, allowing the selection of the most effective treatment strategy for a particular disease. Moreover, it can be used in conjunction with traditional cancer treatment to increase the efficiency of the therapy. Tumor recurrence probably will not happen after immunotherapy due to the formation of memory cells, which carry out the function of immune surveillance, recognizing tumor cells if they start to grow again and eradicating them. However, these benefits come with a risk associated with overactivation of the immune system, which might attack other organs or even lead to the development of autoimmune disease. It should also be borne in mind that the effectiveness of immunotherapy strongly depends on the characteristics and state of the immune system of the individual patient and could decrease in the case of a congenital, acquired, or disease-induced immunodeficiency state. This review examines the interaction of immunostimulatory nucleic acids with the immune system and their potential application for nonspecific immunotherapy of tumoral diseases.

## 2. Recognition of Nucleic Acids and Immunostimulation

### 2.1. Ligands and Receptors

The presence and localization of infectious microorganisms are detected in mammalian cells by pattern recognition receptors (PRRs) [15]. These receptors are ligand-specific sensors that are able to recognize both pathogen-associated molecular patterns (PAMPs) [16] and damage-associated molecular patterns (DAMPs) [17] to orchestrate an early host defense against an infection or injury. Exogenous nucleic acids are one of the PAMP classes, and their molecular features, such as length, double- or single-strand configuration, modification of nucleosides, and sequence motifs, play a key role in immune recognition [18]. These properties—their unusual combinations and abnormal nucleic acid localizations—make it possible to distinguish foreign nucleic acids from endogenous ones.

DAMPs are endogenous host-derived danger signals that are released by damaged or dying cells or upon cellular stress to the extracellular or intracellular space, promoting inflammation in order to clean the tissue from debris for subsequent regeneration [19,20]. The most studied DAMPs include mono- and polysaccharides (glycans) [20], high-mobility group box 1 (HMGB1) [21], nucleic acids [22], and secreted ATP [23].

There are several types of PRRs that sense nucleic acids in mammalian cells, which are located in different cell compartments—on the plasma membrane, in endosomes, and in the cytoplasm—which allows sensors to detect both PAMP and DAMP nucleic acids. The first one is the endosomal subfamily of TLRs (TLR3, 7, 8, and 9). Another type includes cytosolic RNA-binding proteins such as retinoic acid-inducible gene I (RIG-I), melanoma differentiation-associated protein 5 (MDA5), and laboratory of genetics and physiology protein 2 (LGP2). Another type of recently described sensor is cytoplasmic DNA sensors, which are DNA-dependent activators of IRFs (DAI) [24].

The location in endosomes and lysosomes determines the specialization of TLR receptors to recognize foreign nucleic acids that undergo endocytosis after the destruction of the infectious agent or lysis of the infected cell. Each endosomal TLR is able to recognize a specific type of nucleic acid: dsRNA activates TLR3 [25], non-self ssRNA triggers TLR 7 and TLR8 [26], and CpG DNA triggers TLR9 [27]. TLR3 is expressed in myeloid dendritic cells [28]; therefore, it connects the innate and adaptive immune systems, and the other endosomal TLRs are expressed in a wider variety of immune cells, including pDCs [29], macrophages, monocytes, and lymphocytes [30].

Long dsRNA is recognized by TLR3 because it has two non-sequence-specific dsRNA binding sites [31]. In vitro studies have shown that the minimal length of dsRNA required to activate TLR3 is about 40 bp [32].

TLR7 and TLR8 are homologous receptors for ssRNA [33,34]. Both have two binding sites in their leucine-rich domain; however, the specificity of sites in the composition of different receptors differs: the first site binds nucleosides with a preference for guanosine and uridine by TLR7 and TLR8, respectively [35,36]. Nucleotide binding is performed by the second site, where TLR8 binds to UG dinucleotide, and TLR7 prefers minimum 3-mers with U located in the second position [36].

TLR9 is a DNA sensor that preferentially binds to DNA molecules containing unmethylated cytosine–phosphate–guanine deoxynucleotide (CpG) motifs and is expressed in innate immune cells, including macrophages and DCs [37]. When CpG DNA is bound to the extracellular domain of TLR9, they form a symmetric complex, and it was shown that CpG DNA binds to both protomers in the dimer of TLR9 [38]. Further study proved that TLR9 contains a binding site that binds DNA with cytosine in the second position starting from the 5′ end, assisting in the activation of TLR9 [39].

Cytosolic RNA-binding proteins or RIG-I like receptors (RLRs) include three members: RIG-I, MDA5, and LGP2. RLRs belong to the SF2 helicase super-family, which are mostly found in the cytoplasm, but some amount of RIG-I is located in the nucleus [40]. RIG-I and MDA5 are signaling proteins, while LGP2 has a regulatory role [41]. These different functions are due to their structural dissimilarities. RIG-I and MDA5 have a similar structure, with a helicase domain in the middle part and a carboxy-terminal domain (CTD) [42]. Both of these domains are able to detect and bind RNA; moreover, they both possess caspase activation and recruitment domains (CARDs), which mediate signal transduction and lead to type I IFN gene expression [43]. LGP2 lacks CARDs, thus it initiates signaling by regulating RIG-I and MDA5 [44]. The regulatory C-terminal domain of LGP2 binds to the dsRNA binding protein PACT, and this complex inhibits the RIG-I-dependent response and activates the MDA5-dependent response. This interaction allows the cellular RNA silencing machine to coordinate the innate immune response [45].

The presence of functional RIG-I and MDA5 is necessary for an immune response against viral infection [46]. Due to their preference for RNA binding, it was shown that RIG-I and MDA5 can recognize different segments of the same viral genome [47]. Although these proteins share structural similarities and a downstream conserved signaling pathway, they are activated by distinct RNA species. RIG-I prefers binding with short dsRNA, which is tri-phosphorylated at the 5′ end [48]. In other studies, it was suggested that 5′ monophosphate dsRNA is unable to activate RIG-I and at least 5′-diphosphate dsRNA is needed for successful binding and activation [49]. Moreover, RIG-I can distinguish between 5′-diphosphate and 5′-triphosphate dsRNA [50]. These energetic differences of binding with mono-, di-, or triphosphate enables RIG-I to discriminate between endogenous and viral RNA [42]. On the contrary, MDA5 is activated by long dsRNA, which was confirmed by its activation by poly(I:C), a synthetic mimic of long dsRNA [51]. MDA5 binds with dsRNA through the phosphodiester backbone, which makes this binding non-sequence-specific, and during this interaction, the MDA5 forms a ring-like conformation around the dsRNA [52]. Nucleic acid sensors signal through various pathways when detecting the presence of alien nucleic acids; this means the targeting of specific receptors plays a crucial role in determining the immune response when designing immunomodulatory drugs using therapeutic nucleic acids.

Depending on the activated receptor, the nucleic acid or the ligands will induce a specific immune response, resulting in the induction of pro- or anti-inflammatory cytokines. For immunostimulatory RNA (isRNA)-based drugs, targeting TLRs 3/7/8 and RLRs such as MDA5 and RIG-I like receptor to induce type-I interferons might be a good idea, since these sensors preferably bind with different RNA molecules. However, these receptors also send signals to synthesize proinflammatory cytokines and their overstimulation might cause side effects such as chronic inflammation. Determining the feasibility of the therapeutic use of these drugs will require an evaluation of the influence of the pro- or anti-inflammatory cytokine profile on the therapeutic effect, as well as on the tolerance and safety of use.

### 2.2. Sequence Dependent Immunostimulation

Researchers’ efforts to obtain therapeutic drugs based on nucleic acids (antisense oligonucleotides, siRNAs, ribozymes, mRNAs, and plasmids) have revealed the ability of certain nucleic acids to have an immunostimulating effect similar to the response to a foreign agent [4,53]. These effects (inhibition of cell growth, global changes in gene expression, toxic effects) were first revealed when studying antisense oligonucleotides, which include certain sequence motives [54].

It was found that the presence of non-methylated CpG motifs caused immunostimulation, since they are more common in bacterial DNA and rare in mammalian DNA [55,56] and, therefore, regarded in the vertebrate immune system as a danger signal. The sequence motives are recognized by TLR9 and induce an innate immune response [57,58]. Moreover, CpG oligodeoxynucleotides (ODNs) can activate cells of the adaptive immune system, and due to their immunostimulatory activity they have been studied widely in clinical trials as therapeutic agents against oncological and infectious diseases [59,60].

In particular, an oligonucleotide containing CpG motifs was successfully applied in immunotherapy against human bladder cancer (Table 1) [61]. Treatment of tumor cell culture with different concentrations of CpG ODN reduced the viability of human bladder cancer cells (UM-UC-3 and T24) by inducing apoptosis and promoted the viability of normal, non-malignant human uroepithelial cells (SV-HUC-1). The team of Qi et al. used two different CpG ODNs with CG repeats (1826-CpG and KSK CpG) (Table 1) and studied their effect on A20 lymphoma cells. It was noticed that both ODNs induced apoptosis and cytotoxicity in cancer cells, but KSK CpG was more efficient [62].

CpG ODNs with phosphodiester backbone are not stable inside cells and are weak to nuclease degradation, which results in a weak immune response [56]. When a phosphothioate backbone was used the difference was clear: the ODNs were more resistant and their effect lasted longer [59,63]. Another strategy used to stabilize CpG-ODNs without altering the structure of the sugar backbone is to create sequences rich in guanine to induce the formation of G-quadruplex (G4) structures [63,64]. It was established that the GTCGTT sequence with phosphodiester backbone is recognized by human cells as CpG motif [64]. In the study of Hoshi et al., a sequence containing this motif altering with polyguanosine sequence was designed in order to induce the formation of G-quadruplex structure. Sequences including 0, 2, 4, and 8 guanosine nucleotides were tested and the sequence G4-CpG (Table 1) showed increased resistance to nuclease degradation and cellular uptake and had a seven-fold rise in IL-6 secretion compared to other sequences. It also formed G-quadruplex structure better than other sequences [65], indicating a possible impact of the tertiary structure on immune recognition, although the effect of greater resistance to degradation by cellular nucleases cannot be excluded.

The discovery of RNA interference has sparked tremendous interest in studying the effect of double-stranded RNAs on gene expression [66]. Initially, it was believed that short double-stranded RNAs (siRNAs), mimicking the products of Dicer processing of double-stranded RNAs, do not have an immunostimulatory effect, unlike the original long double-stranded RNAs; however, as experimental data accumulated, it became clear that this was not the case [67,68]. During the studies concerning siRNA it was noted that sequences rich in G- and U-zones was a common feature of immunostimulatory motifs [69]. Further studies showed that the GU-rich motif was the main structure to be recognized by TLR8 [70]. In [71], a 5′-triphosphat siRNA was effective in gene silencing and at the same time activated the RIG-I receptor. Another study used 5′-triphosphate siRNA, which activated RIG-I dependent type-I interferon production and inhibited the replication of hepatitis B virus [72].

Short 22 bp dsRNA, named immunostimulating RNA (isRNA), exhibited antiproliferative activity in different tumor cells in vitro and antitumor and antimetastatic activity in carcinoma and hepatoma cells in vivo [54,73,74], and isRNA induced production of type I IFNs and inhibited the development of influenza infection in mice [75]. The immunostimulatory effect of isRNA was found to be sequence dependent (Table 1): replacement of A and U bases at the 3′ end of one strand was enough to block the immunostimulatory activity of the duplex.

Li et al. injected adult male Kunming mice via tail vein with ssRNA derived from viral genomes (HIV-1 and SARS-CoV) with phosphothioate backbone and GU-rich motifs [70]. The results showed that the ssRNA (Table 1) induced a notable increase in TNFα in the serum of treated mice compared to the control group. Moreover, SARS-CoV ssRNA120 was found to cause a significant rise in the level of pro-inflammatory cytokines in human PBMCs.

Notably, replacing U with A in a immunostimulatory sequence prevented stimulation of IL-6 and TNFα production by PBMCs, while replacing G with A blocked only the induction of IFNα in pDC without affecting the induction of IL-6, TNF α, and IL- 12 secretion [73]. When an ssRNA was designed to contain CpG motif and a 6 nt poly-(G) at the 3′ end, it was able to activate monocytes, but the receptors responsible for recognizing this molecule are still unknown [74]. DNA-RNA hybrids were designed in [76] and the findings indicated that it could effectively activate TLR7, TLR8, and TLR9 in vivo, if CpG motifs were included.

Spherical nucleic acids (SNAs) represent an attractive new agent in the field of immunostimulating nucleic acids due to a number of superior properties. First of all, the spherical shape protects them from nuclease degradation as compared to linear nucleic acids, which increases their lifetime inside cells, ensuring a longer duration of biological effect [77]; it also facilitates cellular uptake. SNAs are more tolerated by cells than linear nucleic acids with the same sequences, and their ability to activate the innate immune system depends entirely on the presence of immunostimulating motifs in their sequence, which means they can serve multiple purposes [78]. SNAs can be obtained from either DNA or RNA or their combination by organizing them around a nanoparticle core [79]. SNAs are able to enter the cell without special delivery vehicles through endocytosis, which provides endosomal localization and facilitates their interaction with TLRs [80].

The team of Radovic-Moreno et al. developed a 3D structure of immunostimulating SNAs using different nanoparticles as the core and used CpG 1826 (Table 1) with phosphodiester and phosphothioate backbone. The results showed that the 3D structure of IS SNAs decreased the growth rate of cancer cells and enhanced the survival of lymphoma model animals [79]. Additionally, IS-SNAs led to more pronounced activation of innate immune cells in vivo and enhanced humoral and cellular immune responses to model antigens like ovalbumin (OVA). When the immunostimulatory effect of SNAs made of ssRNA instead of DNA (Table 1) was studied, the data showed that the SNAs entered both antigen-presenting cells (APCs) and non-APCs and caused a notable and sequence-specific activation of TLR7 and TLR8. Furthermore, the conformation of the nucleic acids, which can be controlled while designing the structure, can affect the activity [78].

The acquisition of immunostimulatory patterns can impart appropriate properties to therapeutic nucleic acids aimed at activating or inhibiting the expression of specific genes. A multifunctional molecule consisting of synthetically linked double-stranded siRNA and a single-stranded CpG oligonucleotide agonist of TLR9 was the focus of work by Kortylewski et al. [81]. This conjugate was capable of activating TLR9, targeting specific immune cells (B cells and DCs, key components of the tumor microenvironment), in addition to its immune checkpoint silencing function. The addition of triphosphate to the 5′-end of siRNA targeting the fusion region of S and P genes of the HBV genome increased its immunostimulatory ability by activating intracellular receptor RIG-I and an enhanced antiviral effect [72].

Thus, identifying the immunostimulating motifs of the sequence and structure can allow us not only to avoid including them in the composition of gene-targeted therapeutic nucleic acids, but also to create immunostimulating molecules based on them with a favorable spectrum of activation of cytokines and interferons and provide a balance between immunostimulation and toxicity.

**Table 1:** Experimentally studied immunostimulating nucleic acids.

Type	Sequence 5′–3′	Length n/bp	Effects	Reference
G4-CpG	GGGGTTGTCGTTTTGTCGTTGGGGTTGTCGTTTTGTCGTTGGGGTTGTCGTTTTGTCGTTGGGG	64	Forms G-quadruplex; induces IL-6	[65]
CpG ODN	AACGTTGTCGTCGACGTCGTCGTC	24	Reduces viability of human bladder cancer cells (UM-UC-3 and T24)	[61]
1826-CpG	TCCATGACGTTCCTGACGTT	20	Induces apoptosis in A20 lymphoma cells, but not as effective as KSK-CpG	[62]
KSK-CpG	TCGTCGTTTTCGTCGTCGTTTT	22	Decreases mitochondrial membrane potential; induces apoptosis in A20 lymphoma cells	[62]
ssRNA40 from HIV-1 genome	GCCCGUCUGUUGUGUGACUC	20	Induces TNF-α secretion in mice	[70]
ssRNA120 SARS-CoV genome	GUCUGAGUGUGUUCUUG	17	Induces TNF-α secretion in mice; induces pro-inflammatory cytokine release in hPBMCs	[70]
ssRNA83 SARS-CoV genome	GUGCUUGUGUAUUGUGC	17	Induces TNF alpha release in mice	[70]
ssRNA-DR	GCCCGACAGAAGAGAGACAC	20	Activates TLR 7/8	[78]
short dsRNA	GUGUCAGGCUUUCAGAUUUUUU/ AAAUCUGAAAGCCUGACACUUA	22	Has antiproliferative effect against tumor cells	[73,74]
1826 CpG SNA	TCCATGACGTTCCTGACGTT	20	Decreases growth rate of cancer cells; activates innate immune cells in vivo	[79]

### 2.3. Sequence-Independent Immunostimulation

Characteristics of nucleic acids such as length, duplex structure, and degree of end phosphorylation are decisive in determining “friend or foe” by pattern recognition receptors (Table 2). Long dsRNA, normally not found in mammalian cells, is typically associated with viral infection, and it represents the genetic material of some viruses, or an intermediate state produced during viral replication [82]. The ability of the immune system to respond to a viral infection by synthesizing interferons and cytokines and mobilizing immune cells to the site of infection inspired researchers to use polyriboinosinic:polyribocytidylic acid (poly(I:C)), a synthetic dsRNA that mimics the effects of dsRNA of natural origin as a potential antitumor and antiviral drug [83]. Mismatched double-stranded RNA:polyI:polyC12U (Ampligen) has been used in some countries for the treatment of chronic fatigue syndrome but has not yet received EU or FDA approval due to unwanted side effects [84]. During the last few decades, researchers have been extensively investigating the immune-stimulatory properties of poly(I:C) and the possibility of using it as a vaccine adjuvant [85]. Recently, interest in this drug has reappeared in connection with the COVID-19 pandemic, and AIM ImmunoTech (Philadelphia, PA, USA) initiated a study of Ampligen as a potential infusion treatment for post-COVID-19 cognitive dysfunction.

Heterogeneous synthetic dsRNAs and dsRNAs from natural sources have also been studied as activators of innate immunity. Long double-stranded RNA (472 bp) homologous to the mRNA sequence of *c-MYC* gene was used to achieve silencing of expression of interferon-sensitive *c-MYC* gene by long dsRNAs at two levels of regulation: through the RNAi mechanism and through nonspecific interferon response [86]. It was demonstrated that dsMYC more effectively silenced *c-MYC* expression than dsEGFP (homologous to site 1–448 nt of *EGFP* mRNA) and poly (I:C). dsRNA from virus-like particles from a killer strain of yeast (Ridostin) demonstrated interferon-inducing, phagocytosis-activating, antitumor, and antiviral effects and was approved for clinical use in the Russian Federation as an immunomodulator [87].

The 5′-triphosphate end of ssRNA synthesized by viral polymerases is responsible for activating RIG-I in response to foreign ssRNA [88]. In eukaryotes, detection of the 5’-triphosphate end is revoked by capping it or by post-transcriptional modification. The modification helps the cell to distinguish between host and viral RNAs [89]. Furthermore, dsRNA that was chemically synthesized without phosphate at the 5′-end was able to cause stimulation of immune cells through RIG-I. This indicates that dsRNA can mediate the induction of IFNs via the RIG-I pathway even in the absence of the phosphate group, with the difference being the amount of IFNs, and it was noticed that the presence of the phosphate group enhanced recognition by RIG-I [90].

The length of the nucleic acids strand determines which receptor it will activate: long dsRNA activates MDA5 signaling [52], while short dsRNA is the ligand for RIG-I signaling [91]. It was proven experimentally that shortening poly (I:C), which is an MDA5 ligand, converted it to RIG-I ligand [90]. As another example, in infection by a reovirus, the reovirus RNA genome possesses different fragments of dsRNA with different lengths [92]. Long dsRNAs induce IFNs through the MDA5 pathway, while short dsRNAs cause IFN synthesis via the RIG-I pathway. This means that both RIG-I and MDA5 can discriminate between lengths of dsRNA to start signaling [91]. The specific structure of the ligands of RIG-I can be found in some siRNA structures, such as blunt-ended and in vitro transcribed siRNA containing 5′-triphosphates [68]. LGP2 is also able to participate in the recognition of ss- and dsRNA and bind to RIG-I or MDR5, thus activating signaling [93], and such interaction expands the range of recognized patterns of nucleic acids and the options for responding to them [94].

Circular RNAs (circRNAs) are present in eukaryotic cells and viral genomes. Viral circRNA directly activates RNA pattern recognition receptor RIG-I, and at the same time, N6-methyladenosine (m6A) RNA modification of human circRNAs inhibits innate immunity [95].

Since all cells contain their own DNA in a sufficiently large amount, the detection of foreign DNA is based on the peculiarities of its localization: receptors and proteins located in the cytoplasm such as DNA-dependent activator of IFN-regulatory factors (DAI) [96], absent in melanoma 2 (AIM2) [97], DNA-dependent protein kinase (DNA-PK) [98], and a number of others [99] act as sequence-independent sensors. In the normal state, DNA is absent in the cytosol, when DNA molecules are leaked from the nuclei due to damage or during cell division, they are targeted by the exonuclease DNase III (Trex1) [100] to prevent the activation of STING signaling and subsequently preventing aberrant inflammation and autoimmunity [101,102]. The cGAS-STING pathway is the main sensor of DNA in the cellular cytosol, it plays a vital role in the innate response to inflammation, cancer, and infections [103,104]. In addition to its role in detecting the DNA of pathogens, STNIG can also detect and discriminate between self-DNA released from dying or cancer cells [105]. Moreover, it was reported that mtDNA was able to activate cGAS-STING pathway [106].

**Table 2:** PRR ligands.

PRR	Location	Ligands	Signaling	Reference
TLR 3	Endosome	long dsRNA (minimum length 40–50 bp); poly (I:C)	TRIF-dependent	[34]
TLR 7	Endosome	ssRNA with preference for 3-mers with U located in second position	MyD88-dependent	[107]
TLR 8	Endosome	ssRNA with preference for UG dinucleotides	MyD88-dependent	[34]
TLR 9	Endosome	non-methylated CpG DNA; spherical nucleic acids containing CpG motifs	MyD88-dependent	[58,108]
RIG-I	Cytosol	short dsRNA and ssRNA with 5′-triphosphate; circRNA	MAVS-dependent	[43,49]
MDA5	Cytosol	long dsRNA; poly (I:C)	MAVS-dependent	[43,109]

### 2.4. Signaling Pathways

**Figure 1:**
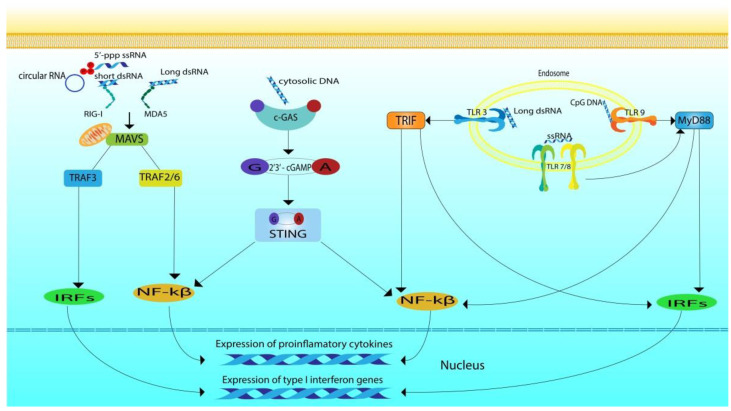
Nucleic acid recognition (drowning was created using Adobe Illustrator (Adobe Inc., 2021, San Jose, CA, USA). Available online: https://adobe.com/products/illustrator, accessed on 21 November 2021).

Intracellular TLRs activate the main pathways mediated by TIR domain-containing adaptor-inducing interferon-β (TRIF) and myeloid differentiation primary response 88 (MYD88) [110] (Figure 1). TLR3 uses the TRIF pathway, which is associated with IFN I and cytokine synthesis [111]. Upon the activation of TLR3, the interaction between TIR-domain-containing adapter-inducing interferon-β (TRIF) and TNF receptor-associated factor (TRAF3) leads to the activation of interferon regulatory factor 3 (IRF3) by phosphorylation through the TRAF3-TBK1-IKKε axis. Activated IRF3 forms a dimer and moves from the cytoplasm into the nucleus, where it prompts expression of IFN-I [108]. TRIF may also interact with TRAF6, which recruits receptor interacting protein kinase 1 (RIPK1). RIPK1 subsequently activates nuclear factor kappa-light-chain-enhancer of activated B cells (NF-kB) and mitogen-activated protein kinases (MAPKs) via TAK1 and induces proinflammatory cytokines [109].

TLRs 7/8/9 signal through the MyD88-dependent pathway, which mostly induces proinflammatory cytokine production [110,112]. MyD88 recruits several interleukin receptor-associated kinases (IRAK1, 2, and 4), which subsequently phosphorylate and activate TRAF6, initiating the ubiquitination and phosphorylation of transforming growth factor β-activated kinase-1 (TAK1). TAK1 primes autophosphorylation of IKKβ [113], which eventually leads to NF-κB activation, then translocation to the nucleus and induction of proinflammatory gene expression [112].

RLR signaling is carried out with the participation of the aspirin domains, which become exposed for interaction with the downstream components of the signaling pathway during conformational changes induced by binding with RNA [114]. After activation of RLRs, the CARD domains of RIG-I and MDA5 become available for interaction with the CARD domains of mitochondrial antiviral signaling protein (MAVS, also known as IPS-1, VISA, or Cardif), which is typically located on the outer membrane of the mitochondria [43]. Activated MAVSs transduce the signal to TRAF3, TBK1 kinase, and IKKε complex. This is followed by phosphorylation of IRF3 and IRF7, which translocate into the nucleus to induce IFN-I synthesis. The produced interferons bind to type I IFN receptors on the membrane of the cell that produced them, and on other cell types that express the same receptors, leading to the activation of Janus kinase/signal transducers and activators of transcription (JAK-STAT) signaling. Eventually the induction of hundreds of interferon-stimulated genes (ISGs) that amplify the IFN response occurs. Activated MAVSs can also pass TRAF2/6, receptor interacting protein-1 (RIP1), and caspase 8/10 pathways, transducing signals to IKK complexes (including IKKα, IKKβ, and IKKγ) and eventually causing phosphorylation of NF-κB and IκBα complexes. Phosphorylated IκBα and activated NF-κB move to the nucleus to promote the production of pro-inflammatory cytokines and inflammatory chemokines.

As for the signals from numerous cytosolic sensors of foreign DNA, stimulator of interferon genes protein (STING) plays an important role in their transmission [115]. In the absence of DNA, cGAS is inactive in the cell [116], when it binds DNA a conformational transition occurs in the active cite, catalyzing the synthesis of the cyclic GMP-AMP (cGAMP) from ATP and GTP [117], followed by the formation of an isomer called 2′3′-cGAMP which serves as a second messenger. 2′3′-cGAMP binds to the endoplasmic-reticulum (ER)-membrane adaptor STING [118,119] causing the activation of STING. STING is expressed in a wide variety of cells including T cells, macrophages, endothelial cells, DCs, and fibroblasts [118,120,121,122]. It detects Cyclic Dinucleotides (CDNs) including c-di-GMP or c-di-AMP from invading bacteria or from cyclic-GMP-AMP synthesized in the cell [119,123]. In unstimulated cells, STING is usually located in the endoplasmic reticulum, it is presented in the form of dimer with its C terminus in the cytosol [123]. Upon activation the C-terminus of STING activates the kinase TBK1, leading eventually to the phosphorylation of IRF3 and its translocation to the nucleus [124]. STING also activates IKK initiating NF-kB translocation to the nucleus [118] where it participates in the regulation of expression along with IRF3 and induces the synthesis of interferons and inflammatory cytokines (Figure 1). ER-associated STING binds to TANK-binding kinase 1 (TBK1) and translocates it to endolysosomal compartments, where TBK1 phosphorylates IRF3 and NF-κB. Activation of the IRF3 and NF-κB signaling pathways leads to the induction of cytokines and type I interferon synthesis.

Thereby, different nucleic acids activating different signaling pathways induce the synthesis of pro- and anti-inflammatory cytokines. The specificity of the ligand–receptor interaction determines the spectrum of resulting effects upon activation. In case of therapeutic nucleic acids, secretion of anti-inflammatory cytokines and type I INFs is preferable, since pro-inflammatory cytokines, including TNF-α, may cause high toxicity and initiate acute or chronic inflammation.

## 3. Challenges and Further Studies

### 3.1. Immunostimulating Nucleic Acids in Cancer Therapy

Nucleic acid-based immunotherapeutics have received increased attention during recent decades [125]. Recently, researchers have concentrated on using therapeutic nucleic acids for cancer therapy by inducing tumor antigen-specific adaptive immune responses, delivering tumor-related antigen with an adjuvant to antigen-presenting cells (APCs), which will induce a tumor-specific immune response [126]. Since a limited number of patients responded to the immunotherapy and undesired side effects such as toxicity and the development of resistance to the therapy were documented [127], it was notable that combining therapeutic nucleic acids as components of non-specific immunotherapy with specific immunotherapy could improve the results in large numbers of cancer patients, even in advanced stages [128]. Cancer cells have developed numerous mechanisms to evade immunosurveillance [129]; therefore, overcoming cancer-induced immunosuppression is critically important for therapy. Approaches aimed at immune checkpoint inhibitors [130] and general stimulation are being actively explored to achieve this goal. PAMPs and other danger signals that activate the innate immune response through specific receptors, including TLRs and cytoplasmic PRRs [131], prompted the idea of mimicking PAMPs to induce an anti-tumor immune response. Nucleic acids can be used for this purpose as the ligands of endoplasmic TLRs [132].

CpG ODNs are intensively studied in pre-clinical and clinical trials as TLR9 ligands (Table 3) [133]. Chemical modifications of CpG-ODNs were successfully used to improve their stability (see above). Signaling through TLR9 causes the induction of cytokines and the activation of APCs [134]. After pre-clinical studies, the results motivated researchers to start clinical trials in the 2000s, testing CpG ODNs alone and accompanied by radio- and chemotherapy, but the outcomes did not match the expectations [135]. Nowadays, CpG-ODNs are tested with immune checkpoint inhibitors in phase 1 and 2 clinical trials for treating advanced tumors such as metastatic melanoma [135].

The most studied nucleic acid inducer of immunity is poly(I:C), a TLR3 ligand [136]. Poly(I:C) was proven to initiate apoptosis in cancer cells [137] and to induce type-I IFN production and chemokine secretion by immune cells [126]. In addition to the immunostimulating effect of dsRNA, it also has an antiproliferative effect on various tumor cells, and this also contributes to its antitumor effect. In addition to the synthetic analogue of dsRNA, various long dsRNAs from natural sources were also studied as antitumor and antiviral agents, but they did not demonstrate significant advantages over the synthetic analogue. Since poly(I:C) is sensitive to nuclease cleavage and can degrade shortly after administration, the use of a naked unprotected poly(I:C) does not allow therapeutically significant effects to be achieved [126,127]. Polyriboinosinic:polyribocytidylic acid–polylysine carboxymethylcellulose (poly-ICLC, Hiltonol^®^), a more stable formulation of poly(I:C), was developed [138] and was evaluated in phase 1 and 2 clinical trials. Poly-ICLC was administered alone or together with radiotherapy and cancer vaccines (Table 4) [139]. Currently, short 21–22 bp siRNAs with immunostimulatory motifs [11,53,140,141,142,143,144] and oligoribonucleotides with 5′-terminal triphosphates [75,145,146] are being investigated as potential adjuvants in antitumor and antiviral immunotherapy; however, these molecules have yet not reached the level of clinical trials.

Some cytokines including IL-1β, IL-6, TNFα, and IFN-γ act as endogenous pyrogens interacting directly with the anterior hypothalamus, which coordinates thermoregulation inducing fever [147]. These cytokines are induced in a response to PAMPs and help the immune system to fight bacterial and viral infections [148]. Therapeutic nucleic acids such as CpG-DNA motifs are presented naturally in the bacterial DNA and they induce a pyrogenic effect through the upregulation of TLR9 signaling pathway and as a result the induction of endogenous pyrogens [149]. Poly(I:C) is known for its ability to induce synthesis of type-I interferons such as IFN-α,β [145] but it can also induce IL-6, IL-12, and TNF-α in human and mouse immune cell culture [146] and these cytokines are known for their pyrogenic activity. When poly(I:C) is administrated systematically it causes different symptoms resulted in fever and sickness behaviors in different species [150]. Chronic inflammation is accompanied with fever and characterized by the constant activation of macrophages and lymphocytes in the infected tissues and with increased levels of IL-1β, IL-6, IL-17, and TNF-α [151]. This gradually disrupts homeostatic interactions between epithelial, stromal, and immune cells in the tissue microenvironment inducing organ fibrosis due to the transition of resident fibroblasts, stellate cells, or fibrocytes into myofibroblast-like cells [152]. Inflammation may not only contribute to the tumorigenesis through the enhancement of growth, survival, and resistance to chemotherapy of cancer cells [153] but also may play an important role in the metastasis of various types of cancer [154]. This indicates the dual role of fever in various pathological conditions and indicates the importance of controlling the spectrum and the duration of effects produced during the activation of innate immunity.

### 3.2. Nucleic Acid-Based Vaccines and Adjuvants

One of the most promising approaches for TNAs is nucleic acid-based vaccines. In typical vaccines, antigen is presented in the form of peptide co-delivered with adjuvants, which will trigger danger signals through APCs [155]. However, there are several obstacles, such as the uptake of the vaccine by regulatory immune cells, which can cause immune tolerance of the tumor, and limited presentation of the antigen by major histocompatibility complex-I, resulting in a weak response by killer T cells [126]. Nucleic acid-based vaccines either encapsulated in lipid nanoparticles or using a vector platform have shown promising results and can be considered as an alternative to conventional vaccination approaches [156]. The use of nucleic acid-based vaccines makes it possible to reduce the time required for their development and promptly respond to emerging challenges. Adjuvants are components of a vaccine that enhance the immunogenicity of the antigen and increase the strength and duration of the immune response by activating the innate immune system. The role of innate immune system activation through pattern recognition receptors (PRRs) in the action of adjuvants of both individual components of the vaccine and those contained in the vaccine itself has been proven [157]. Therefore, immunostimulatory nucleic acids, which are agonists of PRRs, are being actively investigated as vaccine adjuvants (Table 3 and Table 4). The most commonly used agonists for this purpose are CpG-oligonucleotides (TRL9 agonists), dsRNA and its synthetic analogue poly(I:C) (TLR3 agonists), and recently discovered circular RNAs that have been shown to be agonists of RIG-I [95]. The results of Ahn et al. [102] showed that double-stranded DNA species or cyclic di-nucleotides acting as STING-dependent adjuvants (STAVs) can be used to activate antigen presenting cells to promote antigen cross-presentation. Moreover, STAVs has an antitumor activity and cells containing STAVs were able to generate immune responses in mice (C57BL/6J) and STAVs containing cells were able to stimulate CD8^+^ T cells and generate type I interferons.

Naked nucleic acids are not stable in the blood or body fluids or inside cells due to nuclease degradation, and in order to deliver them to endosomes, where nucleic acid sensing TLRs are located, they should be protected, thus encapsulation was used to form lipopolyplexes [158]. Another approach to ensure the integrity of adjuvants and their delivery to target cells is to express them directly in the cells via a viral vector. This approach, in particular, is implemented in the vector platform developed by Vaxart (Table 4), although it should be noted that so far, the drugs created using this technology have not passed beyond phase 2 clinical trials. Nevertheless, the possibility of using nucleic acids as adjuvants looks promising, especially in light of their nontoxicity and safety, which compare favorably with adjuvants based on aluminum salts.

## 4. Conclusions

The recognition of nucleic acids by the immune system for protection against infection by foreign agents is provided by complex evolutionarily conservative mechanisms, the implementation of which involves numerous sensors with different specificities. Such foreign agents include therapeutic nucleic acids introduced into the body from the outside. In the case of specific therapeutic nucleic acids aimed at regulating the expression of specific target genes or editing the genome, immunostimulation is considered an undesirable effect that must be avoided by chemical modification and exclusion of immunostimulatory sequences from their composition. On the other hand, immunostimulation can be effectively used for the purposes of providing immunotherapy for tumor and viral diseases, correcting immunodeficiency states, and increasing the effectiveness of immunization as an adjuvant. Immunostimulatory nucleic acids demonstrate promising results when combined with chemo- or radiotherapy in cancer treatment, but to date, they have not shown sufficient efficiency when used as monotherapy; therefore, this area requires more in-depth research. As for other therapeutic nucleic acids, it is extremely urgent to develop methods for the targeted delivery of immunostimulatory nucleic acids to cells in vivo, since most of the sensors that recognize them are located inside cells. The next stage of both experimental and clinical research will probably be to focus on developing optimal delivery systems and new nucleic acid sequence patterns that can provide a balanced immune response accompanied by a favorable cytokine expression profile. The strategy of combining immunostimulatory nucleic acids with other nucleic acids and non-nucleic acid drugs in order to obtain multifunctional drugs or preparations could increase treatment efficacy and specificity.

## Figures and Tables

**Table 3:** Ongoing and completed clinical trials exploring CpG oligonucleotides.

No.	Interventions	Conditions	Phase	Status	NCT Number
1	CpG-STAT3 siRNA CAS3/SS3 radiation therapy	Recurrent non-Hodgkin’s lymphoma	Phase 1	Recruiting	NCT04995536
2	Anti-OX40 antibody BMS 986,178 TLR9 agonist SD-101	Solid neoplasms	Phase 1	Active	NCT03831295
3	Peptide vaccine GM-CSF TLR9 agonist PF3512676	Stage III–IV melanoma	Phase 1	Completed	NCT00471471
4	Synthetic immunostimulatory DNA conjugated to ragweed allergen	Seasonal allergic rhinitis	Phase 2	Completed	NCT00346086
5	CpG-ODN in situ release of tumor antigen by interventional ablation or drug-eluting beads	Lung cancer, hepatocellular carcinoma, solid tumors	Phase 1	Recruiting	NCT04952272
6	TLR9 agonist MGN1703	HIV	Phase 1 Phase 2	Completed	NCT02443935
7	TLR9 agonist GNKG168	Leukemia	Phase 1	Terminated	NCT01035216
8	CpG-ODN	Glioblastoma	Phase 2	Completed	NCT00190424
9	Na-GST-1/Alhydrogel^®^ CpG 10104	Hookworm disease	Phase 1	Completed	NCT02143518
10	1018 ISS (CpG ODN) irinotecan cetuximab	Colorectal neoplasms	Phase 1	Terminated	NCT00403052
11	1018 ISS (CpG ODN) Hepatitis B vaccine (recombinant)	Hepatitis B	Phase 1	Completed	NCT00426712
12	IMO-2055 (CpG ODN)	Renal cell carcinoma	Phase 2	Completed	NCT00729053

**Table 4:** Ongoing and completed clinical trials exploring dsRNA as adjuvant.

No.	Intervention	Condition	Phase	Status	NCT Number
1	Viral Vector Vaccine Encoding Avian Influenza H5N1 Hemagglutinin Protein and dsRNA Adjuvan (ND1.1)	Avian influenza	Phase 1	Completed	NCT01335347
2	Adenoviral-Vector Based Seasonal Influenza A Vaccine and dsRNA Adjuvant (VXA-A1.1)	Influenza	Phase 1 Phase 1 Phase 2	Completed Completed Completed	NCT01688297 NCT03121339 NCT02918006
3	Adenoviral-Vector Based Norovirus Vaccine Expressing GI.1 VP1 and dsRNA Adjuvant (VXA-G1.1-NN)	Norovirus gastroenteritis	Phase 1 Phase 1 (high dose)	Completed Completed	NCT03125473 NCT02868073
4	Adenoviral-Vector Based RSV F Protein Vaccine and dsRNA Adjuvant (VXA-RSV-f)	Respiratory syncytial virus (RSV)	Phase 1	Completed	NCT02830932
5	Adenoviral-Vector Based Vaccine Expressing a SARS-CoV-2 Antigen and dsRNA Adjuvant (VXA-CoV2-1)	COVID-19	Phase 1 Phase 2 Phase 2 (w/Isotretinoin) Phase 3 (w/Isotretinoin)	Active Recruiting Not yet recruiting Not yet recruiting	NCT04563702 NCT05067933 NCT04577378 NCT04353180
6	Hiltonol (poly ICLC—Poly(I:C) stabilized with polylysine and carboxymethylcellulose)	Healthy volunteers	Phase 1	Completed	NCT01012700
7	NY-ESO-1 protein; poly-ICLC; montanide	Melanoma	Phase 1 Phase 2	Completed Active	NCT01079741 NCT02334735
